# Supplementary material for: Transcription of intragenic CpG islands influences spatiotemporal host gene pre-mRNA processing
Source: Nucleic Acids Res. 2020 Jul 4;48(15):8349–59. doi: 10.1093/nar/gkaa556 (PMC7470969; doi:10.1093/nar/gkaa556)
Supplement: gkaa556_Supplemental_Files [file gkaa556_supplemental_files.zip › Supplementary data.pdf]

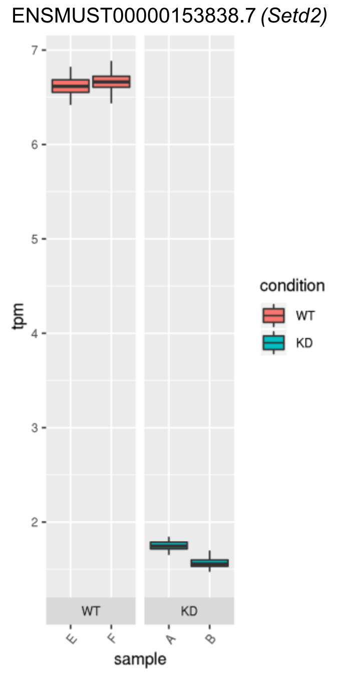

Supplementary Figure S1. Expression of *Setd2* (ENSMUST00000153838.7) in wild type (WT) and knockdown (KD) samples. Data are given as normalised RNA-seq transcript counts (tpm).

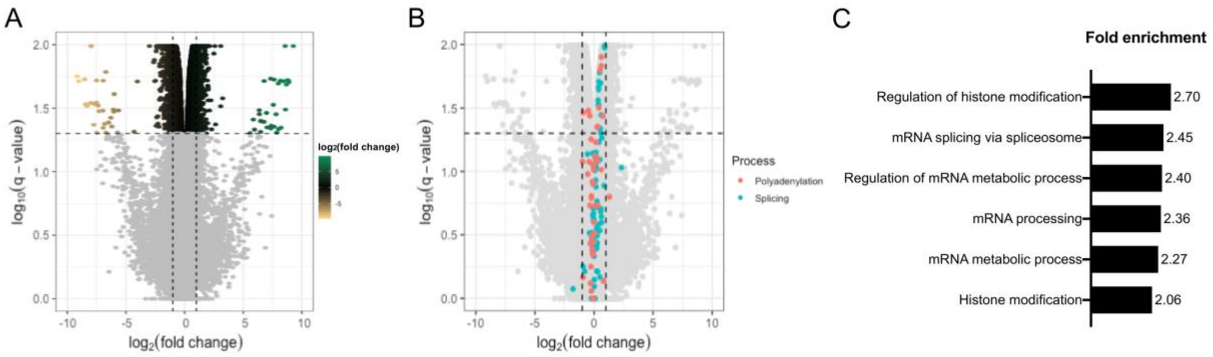

Supplementary Figure S2. (A) *Setd2* knockdown RNA-seq volcano plot illustrating differentially expressed transcripts. Two vertical dashed lines are at log2(fold change) = ±1 and one horizontal dashed line is at q-value = 0.05. (B) Same volcano plot as in A highlighting expression of canonical polyadenylation and splicing factor transcripts. (C) Upregulated biological processes determined by GO analysis (PANTHER). See Supplementary Table S6 for a complete list of GO ID terms.

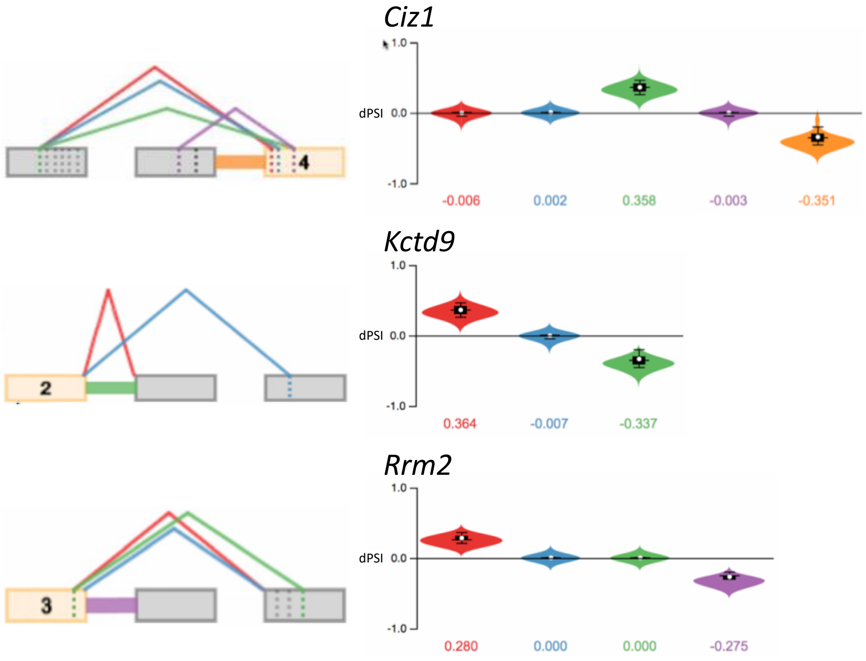

Supplementary Figure S3. Local splice variants (LSVs) plots generated by MAJIQ/Voila. For each gene, all significant LSVs are shown on the left and their associated relative usage values (dPSI) on the right in the same colour. A positive dPSI value indicates that the LSV is used more in *Setd2* knockdown samples and vice versa.

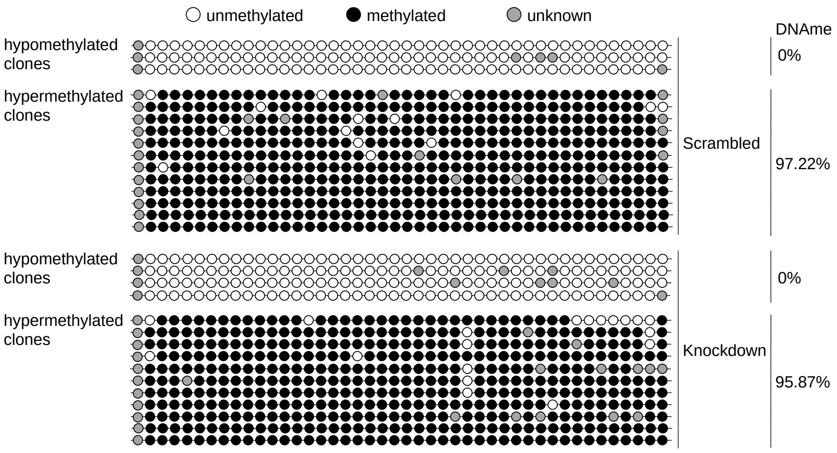

Supplementary Figure S4. *Mcts2/H13* iCGI methylation upon *Setd2* knockdown. Horizontal lines represent individual strands of DNA and circles represent cytosine residues in a CpG context. DNA methylation (DNAme) percentage values are also shown. White circles, unmethylated CpGs; black circles, methylated CpGs; grey circles, the methylation status of the CpG is unknown.

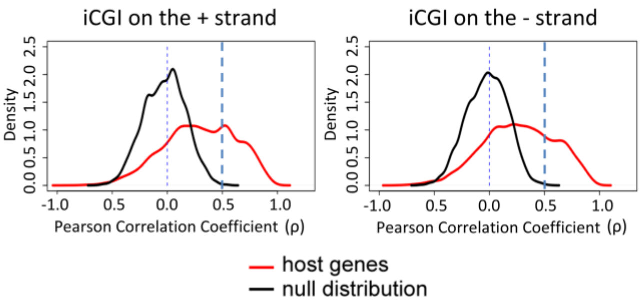

Supplementary Figure S5. Pearson correlation coefficients (ρ) between transcription from the *iCGI* and transcription *upstream:across* the iCGI (see main text and Figure 5A). ρ values were calculated in both sense (left) and antisense (right) orientations with respect to the host gene across 18 human cell lines using RNA-seq data from non-polyadenylated transcripts. A vertical blue dashed line is at ρ=0. A strict cut-off is represented by a second vertical blue dashed line at ρ=0.59, equal to the maximum ρ value observed in the null distribution.

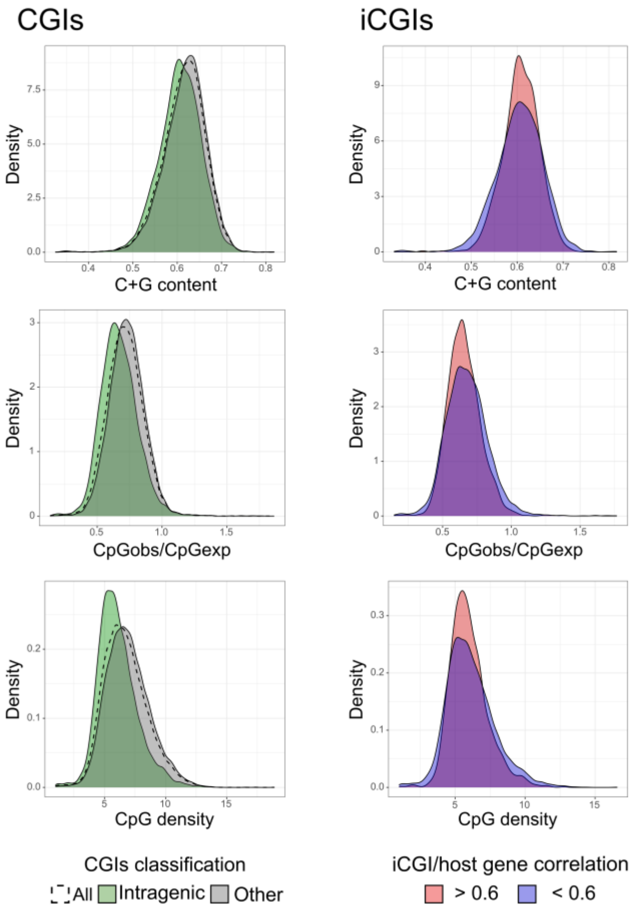

Supplementary Figure S6. Kernel density plots representing the distribution of C+G content (upper), CpG_{obs}/CpG_{exp} ratio (middle) and CpG density (lower) of different CGI classes. In the left panels, CGIs are classified as Intragenic (green) or as Other (grey). In the right panels, iCGIs were segregated according to the Pearson correlation coefficients between transcription from the iCGI and transcription *upstream:across* the iCGI (>0.6 or <0.6).

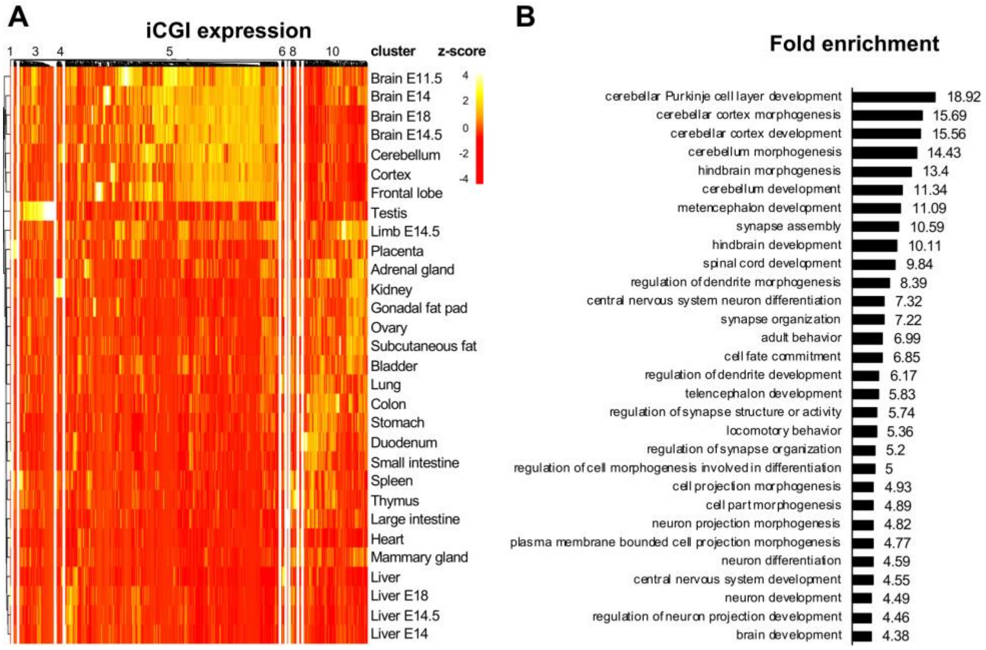

Supplementary Figure S7. (A) RNA-seq heatmap illustrating tissue- and developmental stage-specific transcriptional activity of murine intronic iCGIs within host genes with ρ>0.59 (see Figure 5B). Values are given as column-wise standard-normalised fragments per kilobase of transcript per million mapped reads (z-score). Tissues are from adult mice, unless specified. (B) Upregulated biological processes determined by GO analysis (PANTHER) using host genes harbouring the intronic iCGIs grouped in cluster 5 (see A). See Supplementary Table S8 for a complete list of GO ID terms.

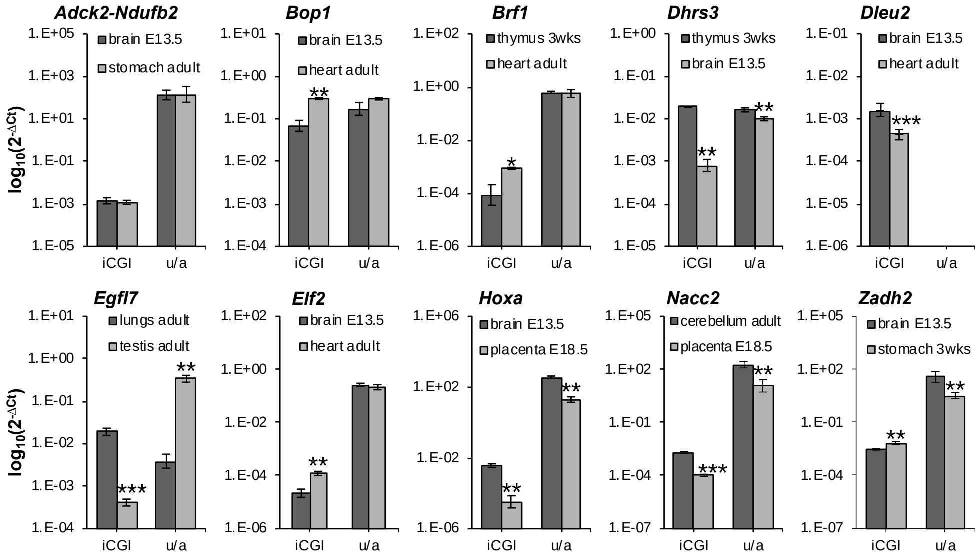

Supplementary Figure S8. mRNA levels of target iCGIs and relative host genes *upstream:across* ratios (u/a) assessed by RT-qPCR. All data are normalised to Ct values for *Actb*. Data are given as $\log_{10}$ of mean $2^{-\Delta Ct}$ values ± 95% confidence interval of three independent experiments. *, p<0.05; **, p=0.01; ***, p<0.001 compared with the other tissue by unpaired *t*-test.

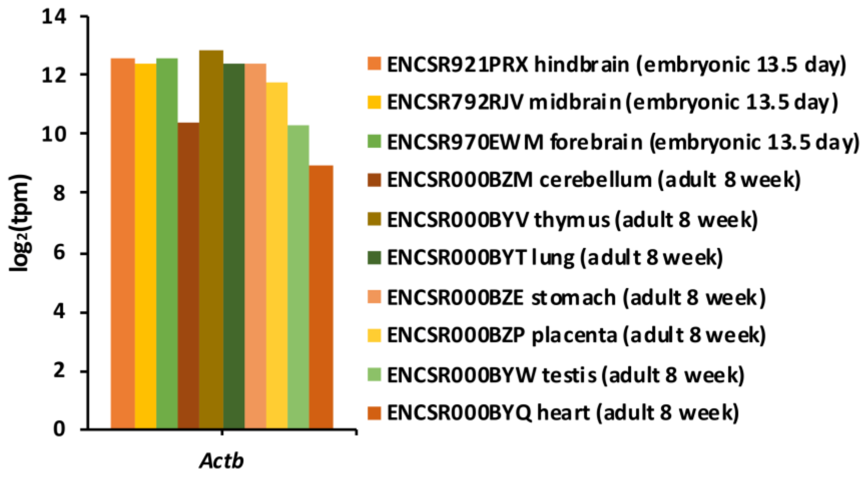

Supplementary Figure S9. *Actb* mRNA levels obtained from the ENCODE website using the SCREEN tool (https://screen.wenglab.org). Counts were normalised to transcripts per million (tpm). Data are given as $\log_2$ of the mean tpm of two technical replicates for each ENCODE biosample. Biosample IDs are indicated in the legend.

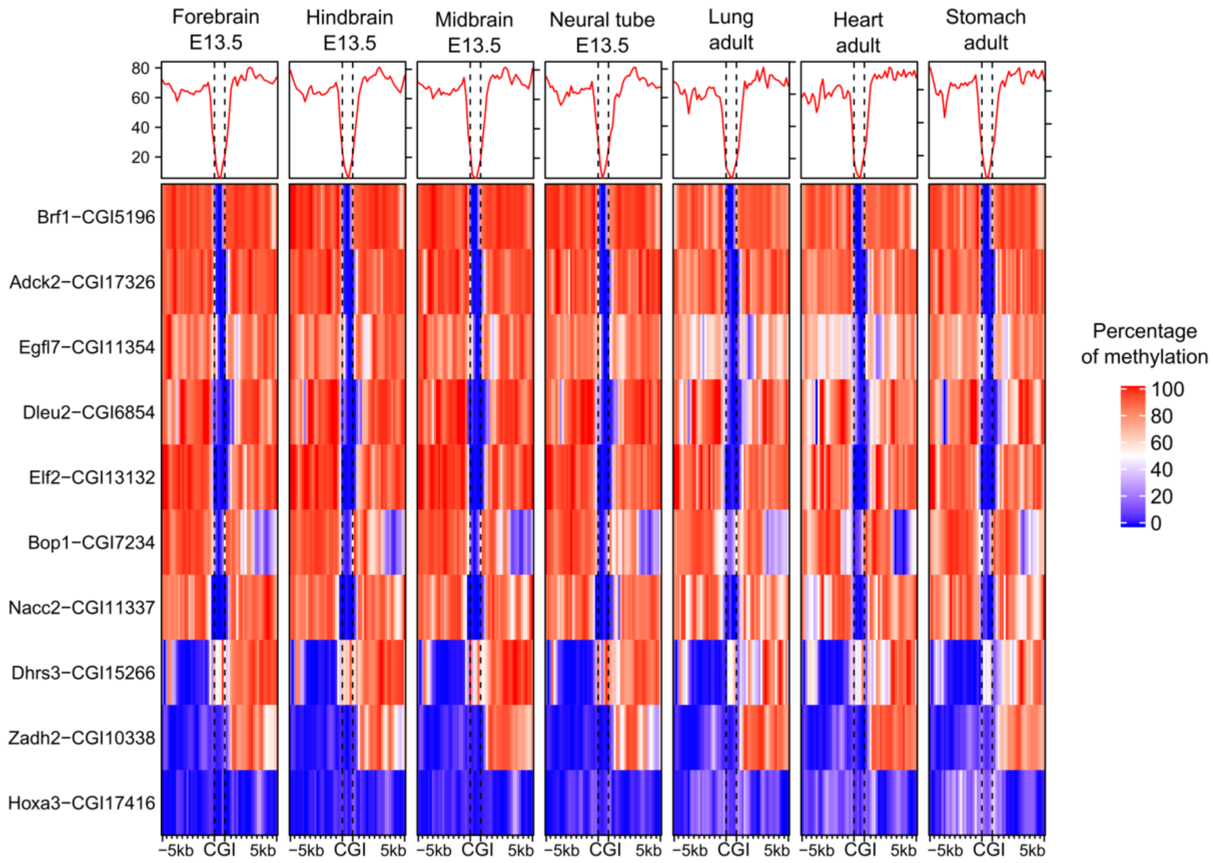

Supplementary Figure S10. ENCODE DNA methylation average percentage plots (upper) and heatmaps (lower) of the ten candidate iCGIs selected after the analysis illustrated in Figure 5. DNA methylation levels are shown at the iCGI and across 5 kb upstream and downstream of it.

Supplementary Table S1. Antibodies

Target	Manufacturer	Cat. No	Dilution
ACTB	Applied Biosystems	8457	1:1000
H3	Applied Biosystems	ab1791	1:1500
H3K36me3	Applied Biosystems	ab9050	1:500
IgG	Applied Biosystems	7074	1:10000

Supplementary Table S2. TaqMan probes

Target	Manufacturer	Assay ID
Actb	Applied Biosystems	Mm00607939_s1
H13	Applied Biosystems	Mm00468785_m1
H13a-c	Applied Biosystems	Mm01241449_m1
H13d	Applied Biosystems	Custom
Mcts2	Applied Biosystems	Mm00481540_s1
Setd2	Applied Biosystems	Mm01250225_m1

Supplementary Table S3. Primers

Target	Forward	Reverse	Experiment
Mcts2 (iCGI)	TTTTTGGTTGTTAAGTATATTTTTGT	TACAATTAAACACACTTTCCTTCTC	1st round nested PCR
Mcts2 (iCGI)	TTTTTAAGTATTAGAATATTGGGGGATT	AACATAATCTTAATAAAAAAACACC	2nd round nested PCR
Elf2 (iCGI)	ACAACAGCTCCGCTCACTTC	TGAAGTAATGGAGGCGACAA	RT-PCR
Elf2 (upstream)	TGAAGTAATGGAGGCGACAA	AGGGAAGGGCAAGGGACT	RT-PCR
Elf2 (across)	CTTGAATCCCTCAAGCAGGT	GTGCTGGTCTATGACGACGA	RT-PCR
Elf2 (iCGI)	GGTTATAGGAAGTGAGAGG	ATCACCTTAAAAAATAACCT	Bisulfite PCR
Nacc2 (iCGI)	GCAGCATCTCAATGAAGCAG	GTCTGTCTTGCACCAACACC	RT-PCR
Nacc2 (upstream)	TGACCTAGGACCACTCAGCA	TGCTGTTCCCTGTGGACATA	RT-PCR
Nacc2 (across)	TGTTCGTTGAGACAGCCAAG	GCTGAGGATGACCTTGAACC	RT-PCR
Nacc2 (iCGI)	TTTAGTTTAGGTAAGTAGAAGG	ACCTACTACCAAACTAAATAAA	Bisulfite PCR
Zadh2 (iCGI)	GCACCATGGACCAAAAGACT	AAACCTGCCAAATCATGACC	RT-PCR
Zadh2 (upstream)	CCCGTCACTTCCTGGACTT	GTTCCGGACGAGGAGGTCT	RT-PCR
Zadh2 (across)	CTGAGCCCTAACTTCCACGA	CCTCACCAATCCCTTCAAAA	RT-PCR
Zadh2 (iCGI)	GTTTTYGGTATTATGGATTAAAAGA	AAACTTTCCATTTCCACCCTCRAA	Bisulfite PCR
Dhrs3 (iCGI)	CAGCTCTTTCTGTGGGAAGG	CACCTGTCCCAAACCAACTT	RT-PCR
Dhrs3 (upstream)	TGCCCTCTCTCTCTCTTTGC	CTGCCTAGCTGCAACCTCTC	RT-PCR
Dhrs3 (across)	GGGAGTCAGTCCTCATCACC	CTTCTCTCGGACAGCTTTGG	RT-PCR
Adck2-Ndufb2 (iCGI)	TGGGAACTGCAGTCTTGCTA	CAGCGCTGCCTGTTCAAG	RT-PCR
Adck2-Ndufb2 (upstream)	TTCCAGCCTCCTATCCAGTG	GAAGGGTTTTGCTGCCTCTA	RT-PCR
Adck2-Ndufb2 (across)	GCCTGCCTGCCTGTATCTAT	ATCCGAGTCATGCCAAAATC	RT-PCR
Adck2-Ndufb2 (iCGI)	TAATGGTTTGAATTTGTTTTTT	ACTAAATTCTTATCACTCRAATCTT	Bisulfite PCR
Brf1 (iCGI)	CCAGTCCCTCTGCAGCTACT	CAGCCATCAGCTCGTTGTT	RT-PCR
Brf1 (upstream)	ATTTTCCGTCCTCGGGTAAG	TGCTCGAGGACAACATCATC	RT-PCR
Brf1 (across)	GTGTGCATCCAATCCCTTTT	GAAGACGTTCCTGCTCTTGG	RT-PCR
Brf1 (iCGI)	GTTAGTTTTTTTGTAGTTATTG	CCCTTACCCACCTTATAA	Bisulfite PCR
Bop1 (iCGI)	CCCAAACAGATCTGCACCTT	GGCTCTCCGTGACTCTTCAG	RT-PCR
Bop1 (upstream)	TCGCTGTCAGAGAGACTGGA	GAAGCGGCAGTAGATCGGTA	RT-PCR
Bop1 (across)	GGGTCATCCATTTTGTCCAG	CAAGGACAGAGGAAGCAGGT	RT-PCR
Bop1 (iCGI)	ATTTTTTTAGGTTAGAAAGGGAG	CAACACATTACCCAAATAAAAAA	Bisulfite PCR
Egfl7 (iCGI)	ACCGAATCTGATCACCGAAC	CCTTCCCACCTACGACTCAA	RT-PCR
Egfl7 (upstream)	AGGCCAGAAGTTCAGTGGTG	GGTACTGGTGGTGGCTGTTC	RT-PCR
Egfl7 (across)	GAACAGCCACCACCAGTACC	TACCATCTGCTGCCAACACT	RT-PCR
Dleu2 (iCGI)	GGGCTATTTAGAGGGCCATT	TTAGAGCAGAGGGCAGTCG	RT-PCR
Dleu2 (across)	TTTCTGCCCGAGAGAGAGAA	GTCTTCCTCCGTCGCTGA	RT-PCR
Dleu2 (iCGI)	GATAAAATGATTTAGTTG	RAAATCTACCCTCACTTT	Bisulfite PCR
Hoxa (iCGI)	AGGTAGCGGTTGAAGTGGAA	ACCCACATCAGCAGCAGAG	RT-PCR
Hoxa (upstream)	AGGTAGCGGTTGAAGTGGAA	ACCGACCGGAAGTACACAAG	RT-PCR
Hoxa (across)	ACCGTAGATCGCTGAGCTGT	GTTGTCGCTGGAGGTGGA	RT-PCR
Hoxa (iCGI)	TAGAGAATAGAGAGTGATTAAGA	AACCCAAATACAACCAACC	Bisulfite PCR
SP6	ATTTAGGTGACACTATAG		Bisulfite sequencing
T7	TAATACGACTCACTATAGGG		Bisulfite sequencing

Supplementary Table S4. Intron retention and splicing changes in *Setd2* knockdown, mutant or knockout sample

	Intronic bases (%)		
**Dataset**	***Setd2* WT**	***Setd2* KD, MT or KO**	**Significant splicing changes**
This paper	0.111 (± 0.00837)	0.0994 (± 0.00705)	136
Simon *et al.* (28)	0.278 (± 0.0758)	0.257 (± 0.0929)	0
Ho *et al.* (29)	0.0632	0.0623	500

The percentage of intronic bases present in *Setd2* wild type (WT) and *Setd2* knockdown (KD, this paper), mutant (MT, Simon *et al.*) or knockout (KO, Ho *et al.*) RNA-seq datasets is calculated as a proxy for intron retention events. No significant changes are observed. The number of significant splicing changes between *Setd2* WT and KD, MT or KO samples is also shown as calculated by MAJIQ/Voila (see Materials and Methods).

Supplementary Table S5. *H13* splice donor sites scores

**Exon**	**Score**	**Exon**	**Score**
1	8.1	7	9.2
2	9.7	8	8.8
3	4.4	9	10.4
4	5.9	10	10.1
5	12.6	11	11
6	8.8	12	8.1

The score expresses how similar the splice sites are to the consensus sequence. A 100% match to the mammalian splice donor site (AAGGTAAGT) would give a score of 12.6. The average score of constitutive splice donor sites is 8.1.
